# Therapeutic potential of recombinant human collagen XVII in blue light-induced skin photoaging: preserving epidermal-dermal structural integrity and functional homeostasis

**DOI:** 10.3389/fbioe.2026.1806274

**Published:** 2026-04-28

**Authors:** Xudong Wang, Linlin Zhang, Qiang Li, Junhong Ao, Xin Zhang, Xin Yang, Jijin Li, Shijia Sui, Ge Ge, Haitao Li, Rongya Yang

**Affiliations:** 1 Department of Dermatology, Seventh Medical Center of Chinese PLA General Hospital, Beijing, China; 2 Medical School of Chinese PLA, Beijing, China; 3 Department of Dermatology, Southern Medical District of Chinese PLA General Hospital, Beijing, China; 4 Department of Dermatology, Shanghai Huamei Aesthetic and Cosmetic Hospital, Shanghai, China; 5 Medical Health Care Department, Air Force Medical Center PLA, Beijing, China

**Keywords:** blue light, fibroblast, keratinocyte, photoaging, recombinant human collagen XVII

## Abstract

**Background:**

Excessive exposure to blue light (BL) contributes to skin photoaging by inducing oxidative stress, inflammation, extracellular matrix degradation, and disruption of epidermal-dermal integrity. Recombinant human type XVII collagen (rhCol17) is a bioactive material with high biocompatibility and reported roles in tissue repair. However, its protective effects against BL-induced photoaging remain unclear.

**Methods:**

*In vitro* and *in vivo* photoaging models were established using human keratinocytes (HaCaTs), dermal fibroblasts (HDFs), and rat dorsal skin exposed to BL. The protective effects of rhCol17 were assessed by Cell Counting Kit-8 assays, quantitative real-time polymerase chain reaction, Western blot, cell migration assays, enzyme-linked immunosorbent assay, immunofluorescence, histopathology, ultrasound measurements, and transmission electron microscopy.

**Results:**

*In vitro*, rhCol17 treatment enhanced BL-impaired HaCaT and HDF viability and migration, while reducing cellular senescence, reactive oxygen species accumulation, and pro-inflammatory cytokine production. *In vivo*, rhCol17 ameliorated BL-induced photoaging, as evidenced by reduced transepidermal water loss, normalization of epidermal and dermal thickness, preservation of collagen, mitigation of elastic fiber degeneration, and increased hemidesmosome density. Furthermore, rhCol17 restored the expression of key collagen and basement membrane components and attenuated MMPs upregulation both *in vitro* and *in vivo*. Mechanistically, rhCol17 modulated Notch signaling in HaCaTs, contributing to the prevention of cellular senescence.

**Conclusion:**

These findings suggested that rhCol17 protects against BL-induced molecular damage and functional impairment in epidermal and dermal cells, while maintaining epidermal-dermal structural integrity. rhCol17 represents a promising therapeutic strategy for preventing BL-related skin damage and offers preliminary insights for potential clinical translation.

## 1 Introduction

Photoaging is a process of severe skin damage induced by solar radiation or other exogenous environmental factors, ultimately leading to premature skin aging (Kaltchenko and Chien, 2025). Clinically, photoaged skin is characterized by wrinkles, laxity, hyperpigmentation, elastosis, and progressive deterioration of skin structure and appearance (Gollogly et al., 2024). Extensive evidence has established ultraviolet (UV) radiation as a major contributor to photoaging (Song et al., 2026; Zhang et al., 2026). However, increasing attention has been directed toward blue light (BL) exposure, which has been reported to induce more persistent hyperpigmentation under certain conditions compared with UV radiation (Duteil et al., 2014). BL (400–500 nm), classified as high-energy visible light, can penetrate the epidermis and reach the dermis. Emerging studies indicate that excessive exposure to BL, particularly within the 400–450 nm range, plays an important role in photoaging (Bacqueville et al., 2022; Das et al., 2023). BL overexposure promotes inflammatory responses and the excessive production of reactive oxygen species (ROS) (Oh et al., 2016; Nakashima et al., 2017), leading to the upregulation of matrix metalloproteinases (MMPs) and subsequent degradation of the extracellular matrix (ECM) (Avola et al., 2019). Although photoaging is largely preventable and partially reversible, effective materials specifically targeting BL-induced skin damage remain limited, highlighting an unmet need in both clinical treatment and daily skincare.

Collagen is a major structural component of the skin ECM and is essential for maintaining skin integrity (Bielajew et al., 2020). During photoaging, collagen homeostasis is disrupted due to decreased synthesis and increased MMP-mediated degradation, resulting in structural weakening of the skin (Brennan et al., 2003). Type XVII collagen (COL17), encoded by the COL17A1 gene, is a transmembrane collagen and a critical component of hemidesmosomes within the basement membrane (BM) zone (Natsuga et al., 2019). Notably, hemidesmosomes decline during skin aging, and COL17 is the only hemidesmosomal component that progressively decreases with age (Dos Santos et al., 2015). COL17 plays a pivotal role in mediating epidermal-dermal anchorage and withstanding mechanical stress, thereby preserving skin integrity (Franzke et al., 2003; Liu et al., 2019). Recombinant human type XVII collagen (rhCol17) has recently emerged as a promising biomaterial with high biocompatibility and low immunogenicity. Compared with native collagen, rhCol17 overcomes limitations associated with high molecular weight and exhibits improved skin penetration potential (Tao et al., 2025). Previous research has demonstrated the anti-inflammatory and tissue repair properties of rhCol17 (Hao et al., 2024). Moreover, rhCol17 has been shown to protect against UV-induced photoaging by preserving BM integrity (Wang J. et al., 2025), suggesting its potential role in mitigating photoaging-associated skin damage. However, the protective effects and underlying mechanisms of rhCol17 in BL-induced photoaging remain largely unexplored.

In the present study, we investigated the protective effects of rhCol17 against BL-induced photoaging using both *in vitro* models (keratinocytes and fibroblasts) and an *in vivo* rat model. We further explored the potential molecular mechanisms underlying rhCol17-mediated protection in keratinocytes. Our results demonstrated that rhCol17 improves the viability of keratinocytes and fibroblasts, restores epidermal barrier function, and attenuates dermal ECM degradation, thereby preventing BL-induced structural damage and skin homeostasis imbalance. Mechanistically, rhCol17 alleviates keratinocyte photoaging through modulation of the Notch1-associated senescence pathway. Collectively, these findings highlight the therapeutic potential of rhCol17 in photoaging and provide new insights for skin damage treatment and daily skincare applications.

## 2 Materials and methods

### 2.1 Cell culture and processing

Human immortalized epidermal cell line (HaCaTs, Cat#06.0150) and primary human dermal fibroblasts (HDFs, Cat#PC.00267) were procured from EallBio (Beijing, China) and grown in their specific culture media (EallBio) in a 37 °C, 5% CO_2_ incubator.

The rhCol17 (molecular weight: 58.2 kDa) was obtained from Lthink Biotechnology (Guangzhou, China). rhCol17 was fully dissolved in ultrapure water and subsequently filtered through a 0.22-μm pore-size membrane. The solution was then diluted with serum-free DMEM (Eallbio, Cat. No. 03.1002C) to final concentrations of 4, 2, 1, and 0.5 mg/mL. Aliquots of rhCol17 (100 μL per well) at the indicated concentrations were added to 96-well plates and incubated overnight at 4 °C. After incubation, residual solution was removed, and the plates were sterilized under ultraviolet light for 30 min and allowed to air-dry under sterile conditions were divided into four groups: control, BL, BL + Col17, and Col17. Cells in the BL + Col17 and Col17 groups were seeded onto rhCol17-coated 96-well plates, whereas cells in the control and BL groups were seeded onto uncoated plates. After incubation at 37 °C, 5% CO_2_ for 24 h, the culture medium was replaced with fresh DMEM. Cells in the BL and BL + Col17 groups were then exposed to 430-nm LED BL at an energy density of 20 J/cm^2^, followed by growth for 24 h at 37 °C after irradiation, and subsequent experiments were performed.

Based on previous research (Zhang H. et al., 2024), HaCaT cells were treated with the ADAM10 inhibitor GI254023X (2 h, 10 μM, MedChemExpress) or the Notch activator Valproic acid (VPA, 24 h, 2 mM, MedChemExpress) following BL irradiation.

### 2.2 Cell viability analysis

HaCaT or HDF cells in the logarithmic growth phase were seeded at a density of 10^4^ cells per well in 96-well plates coated with rhCol17 at concentrations of 4, 2, 1, or 0.5 mg/mL. Following BL irradiation, cells were further incubated for 24, 48, or 72 h. Subsequently, 10 μL of Cell Counting Kit-8 (CCK-8) reagent was added to each well and incubated for 90 min. Absorbance at 450 nm was measured using a microplate reader to evaluate cell viability. Relative cell viability (%) at each time point was calculated and normalized to the control group.

### 2.3 Reactive oxygen species assay

HaCaTs or HDFs were seeded at a density of 10^6^ cells per well in 6-well plates pre-coated with rhCol17 (2 mg/mL). Cells were divided into four groups and treated as described above, followed by incubation at 37 °C for 24 h. Subsequently, cells were incubated with 1 mL per well of DCFH-DA working solution (1:5000; EallBio, Cat#02.10393) for 20 min at 37 °C. After incubation, excess DCFH-DA was removed by washing with PBS. Intracellular ROS levels were assessed by observing fluorescence intensity under a fluorescence microscope.

### 2.4 β-galactosidase senescence assay of cells

HaCaTs or HDFs cultured in 6-well plates were fixed with β-galactosidase staining fixative (Beyotime, China, Cat#C0602) at room temperature for 15 min. After washing with PBS, 1 mL of freshly prepared β-galactosidase staining working solution was added to each well according to the manufacturer’s instructions, followed by incubation at 37 °C overnight. Images were captured using an inverted microscope (Olympus, Tokyo, Japan), and the percentage of β-galactosidase–positive areas was quantified using ImageJ software.

### 2.5 Scratch assay

HaCaTs and HDFs cultured in 6-well plates were subjected to a linear scratch using a sterile pipette tip. After BL irradiation, cells were returned to a 37 °C incubator for continued culture. Images of the wound area were captured at 0, 24, and 48 h. The area of scratch closure was quantified as the percentage of wound area using the formula: Scratch closure (%) = [wound area (0 h) − wound area (t h)]/wound area (0 h) × 100 %.

### 2.6 Transwell assay

HaCaTs and HDFs were seeded in culture plates and serum-starved in serum-free medium for 24 h prior to the experiment. A total of 200 μL of the cell suspension was added to the upper chamber of the Transwell insert (NEST, Cat#725321), while 600 μL of complete culture medium was added to the lower chamber. After overnight incubation, the cells in the upper chamber were treated according to the different experimental groups. After 24 h, the Transwell inserts were transferred to a new 24-well plate and fixed with fixative for 30 min, followed by three washes with PBS. The migrated cells were then stained with 600 μL of crystal violet solution for 15 min and washed with PBS to remove excess dye. After air drying, the membranes were mounted, and five random fields were selected for cell counting under a microscope to evaluate cell migration ability.

### 2.7 Quantitative real-time polymerase chain reaction (qRT-PCR)

Total RNA was extracted from HaCaTs or HDFs cultured in 6-well plates using Unizol reagent. cDNA synthesis was performed with the UnionScript First-strand cDNA Synthesis Mix for qPCR (with dsDNase, EallBio, Cat#04.11400). qRT-PCR was subsequently performed using the GS AntiQ qPCR SYBR Green Fast Mix (EallBio, Cat#04.11500). GAPDH was used as the internal control, and the relative expression levels of inflammatory cytokines, collagen, and extracellular matrix (ECM)-related genes were calculated using the 2^−ΔΔCt^ method. The primer sequences are listed in Supplementary Table S1.

### 2.8 Enzyme-linked immunosorbent assay (ELISA)

HaCaTs or HDFs were collected and centrifuged at 3,000 rpm for 20 min at 4 °C. The supernatant was collected, and IL-1β, IL-6, and TNF-α were detected by the human ELISA kits (Boster, Wuhan, China) according to the manufacturer’s instructions.

### 2.9 Western blot

Total proteins were extracted from HaCaTs or HDFs using RIPA lysis buffer (EallBio, Cat#02.12264) and quantified by the BCA protein assay kit (EallBio, Cat#02.12267). The proteins (25 µg/lane) were separated by 8% SDS-PAGE and transferred to polyvinylidene fluoride membranes. The membranes were blocked with 5% skimmed milk for 2.5 h. Then, the membranes were incubated with primary antibodies against COL4A1 (Boster, Cat#PB9099, 1:750), COL7A1 (Boster, Cat#A01170-1, 1:1000), COL17A1 (Boster, Cat#A03031-1, 1:1000), LAMB3 (ZEN-BIO, Cat#822605, 1:750), MMP2 (ZEN-BIO, Cat#R380817, 1:750), COL1A1 (ZEN-BIO, Cat#R26615, 1:750), COL3A1 (ZEN-BIO, Cat#23957, 1:750), MMP1 (ZEN-BIO, Cat#R383245, 1:750), MMP3 (ZEN-BIO, Cat#R24995, 1:750), Notch1 (Proteintech, Cat#10062-2-AP, 1:2500), HES1 (Boster, Cat#BM4488, 1:1000), SIRT1 (Boster, PB0173, 1:1000), P16 (Boster, Cat#A00016-5, 1:750), P21 (Boster, Cat#A00145-1, 1:750), and P53 (Boster, Cat#PB9008, 1:1000) overnight at 4 °C. After washing, the membranes were incubated with HRP-conjugated secondary antibodies for 1 h at room temperature. Protein bands were visualized using the ECL detection reagent and captured using the Gel Imaging Analysis System (Tanon 5200 Multi). The band density was quantified using analysis software (v 4.2, Tanon QuickChemi 5200 Monad GIS 1D) to determine the relative protein levels normalized against GAPDH.

### 2.10 Animal model establishment

Sixteen male specific pathogen-free (SPF) Sprague-Dawley (SD) rats, weighing 200–250 g, aged 8 weeks, were purchased from Jinmuyang Experimental Animal Co., Ltd. (Beijing, China). Before the experiments, all rats were acclimatized for 1 week under controlled conditions: a temperature of 21 °C ± 2 °C, relative humidity of 55%–60%, and a 12 h light/12 h dark cycle. The SD rats were randomly divided into four groups: Control, BL, NaCl, and rhCol17. Rats in the latter three groups were exposed to BL irradiation for 12 h per day (430 nm, 120 J/cm^2^). For the rhCol17 group, microneedling was performed to create transdermal delivery channels, followed by topical application of 20 mg/mL rhCol17 solution (dissolved in NaCl). The NaCl group received similar treatment but with physiological saline as a control. Skin thickness and physiological parameters were assessed weekly using a skin ultrasound system (50 MHz, Meditec, China) and a multifunctional skin physiology analyzer (CK, Germany) to measure transepidermal water loss (TEWL), respectively. After 3 weeks, rats were euthanized by intraperitoneal injection of pentobarbital sodium (60 mg/kg). Dorsal skin tissues were harvested for histopathological evaluation.

### 2.11 Histopathological staining and evaluation

Skin tissues were fixed in 4% methanol-based fixative and processed into formalin-fixed, paraffin-embedded (FFPE) sections according to standard protocols. FFPE sections (4 μm thickness) were subjected to hematoxylin and eosin (H&E) staining to examine skin morphology and to measure the thickness of the epidermis and dermis. Periodic acid-Schiff (PAS) staining was performed to assess the integrity of the BM. Masson’s trichrome staining was used to evaluate changes in collagen fiber area, while orcein staining was applied to visualize alterations in elastic fibers. All staining kits were obtained from Solarbio (Beijing, China), and the experimental procedures were performed following the kit protocols.

### 2.12 Transmission electron microscopy (TEM)

Fresh skin tissues (∼1 mm^3^) were fixed in pre-cooled electron microscopy fixative (Baiqiandu, China) at 4 °C for 2–4 h, followed by post-fixation with 1% osmium tetroxide in 0.1 M PBS (pH 7.4) at room temperature for 2 h. After thorough washing with PBS, the samples were dehydrated through a graded ethanol series. Tissues were infiltrated overnight with a 1:1 mixture of acetone and Epon 812 resin (SPI, USA), followed by infiltration with pure Epon 812 resin overnight. Polymerization was performed at 60 °C for 48 h. Ultrathin sections (60–80 nm) were stained with 2% aqueous uranyl acetate and lead citrate for 15 min each, then air-dried overnight. Sections were examined under a transmission electron microscope (FEI, USA), and the number of hemidesmosomes was quantified.

### 2.13 Immunofluorescence staining

FFPE sections of rat skin were subjected to antigen retrieval and endogenous peroxidase blocking, followed by permeabilization with 0.5% Triton X-100 for 20 min. After washing with PBS, sections were blocked with 5% goat serum for 60 min at room temperature. The sections were then incubated overnight at 4 °C with primary antibodies against SA-β-gal (Cell signaling, Cat#27198T, 1:100), IL-6 (Abcam, Cat#Ab9324, 1:100), TNF-α (Abcam, Cat#Ab307164, 1:100), COL4A1 (Abcam, Cat#Ab6586, 1:100), COL7A1 (Abcam, Cat#Ab309143, 1:100), COL17A1 (Abcam, Cat#Ab184996, 1:100), MMP1 (Invitrogen, Cat#PA5-102381, 1:100), MMP9 (Abcam, Cat#Ab76003, 1:100), Notch1 (Proteintech, Cat#10062-2-AP,1:100), HES1 (Abcam, Cat#Ab108937, 1:100) and SIRT1 (Proteintech, Cat#RMX00009, 1:100). After three washes with PBS, sections were incubated with secondary antibodies (ZSGB-BIO, Cat#ZF-0311/ZF-0312/ZF-0516, 1:200) for 2 h at room temperature. The sections were subsequently washed with PBS and counterstained with DAPI for 5 min to visualize nuclei. Fluorescence images were captured using a fluorescence microscope, and fluorescence intensity was quantified using ImageJ software.

### 2.14 Statistical analysis

Statistical analyses were performed using GraphPad Prism (v 8.3.0; Dotmatics) and data are presented as the mean ± standard deviation. Statistical comparisons were performed one-way ANOVA followed by Tukey’s *post hoc* test for multiple comparisons.

## 3 Results

### 3.1 rhCol17 alleviated BL-induced photoaging in keratinocytes

**FIGURE 1:**
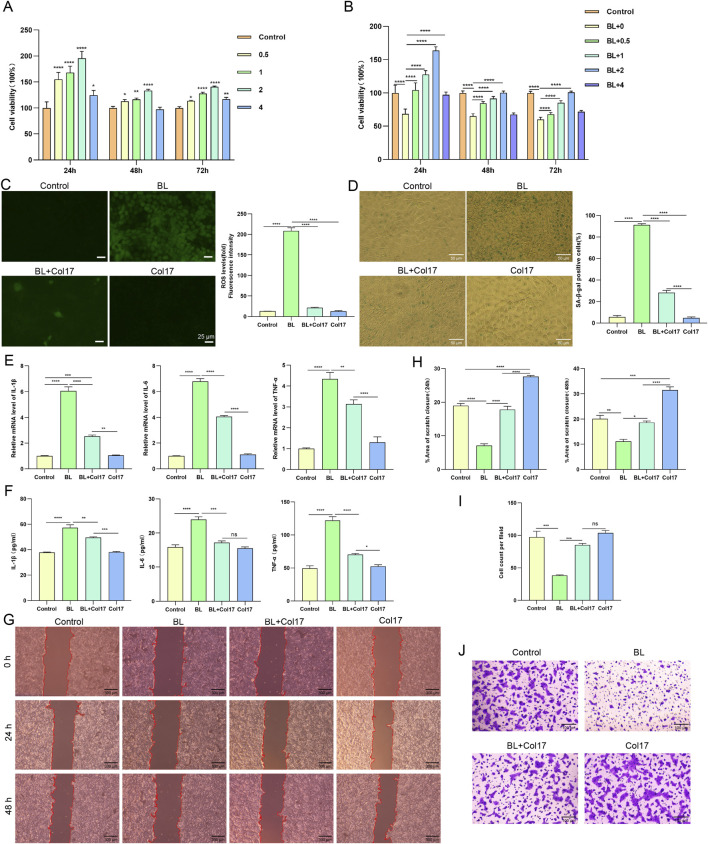
rhCol17 alleviated BL-induced photoaging in HaCaTs. **(A)** Cell viability assessed by CCK8 assay at 24, 48, and 72 h after treatment with different concentrations of rhCol17. P-value vs. control group. **(B)** Cell viability assessed by CCK8 assay at 24, 48, and 72 h after BL exposure with or without rhCol17 pretreatment at the indicated concentrations. **(C)** Representative fluorescence images (left, scale bar = 25 μm) and quantification (right) of cellular ROS levels measured using DCFH-DA assay. From left to right: control, BL exposure group, BL + rhCol17 group (BL exposure with 2 mg/mL rhCol17 pretreatment), and Col17 group (rhCol17 only). **(D)** Representative images (left, scale bar = 50 μm) and quantification (right) of SA-β-gal staining. **(E)** Relative mRNA expression levels of IL-1β, IL-6, and TNF-α determined by qRT-PCR. **(F)** Concentrations of IL-1β, IL-6, and TNF-α in the culture supernatants measured by ELISA. **(G)** Representative images of scratch assays at 24 and 48 h (scale bar = 300 μm). **(H)** Quantitative analysis of scratch closure area (%) in the scratch assay. **(I)** Quantitative analysis of the migrated cell count per field. **(J)** Representative images of transwell migration assays. N = 3. *P < 0.05, **P < 0.01, ***P < 0.001, ****P < 0.0001.

HaCaT cells, which retain the characteristics of epidermal keratinocytes, are widely used for *in vitro* studies of epidermal homeostasis and pathophysiology (Carlin et al., 2025). We first examined the protective effects of rhCol17 on HaCaT cells exposed to BL. CCK-8 assays showed that rhCol17 treatment alone, or in combination with BL exposure, significantly increased HaCaT cell viability in a concentration-dependent manner (0.5–2 mg/mL) (Figures 1A,B). However, no further protective effect was observed at 4 mg/mL. These findings suggested that 2 mg/mL represents an optimal concentration, which was used in subsequent experiments.

**FIGURE 2:**
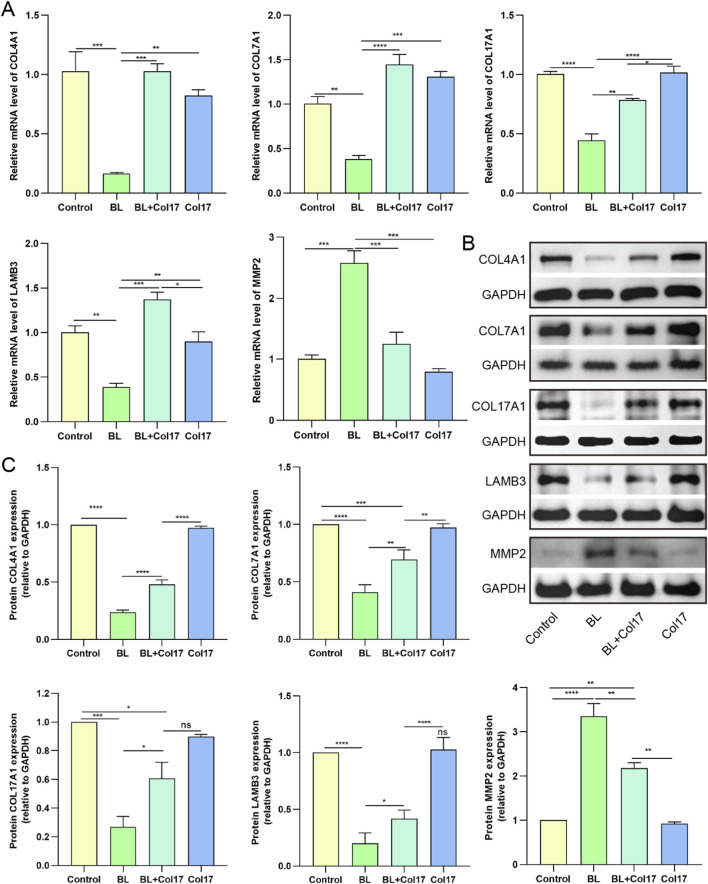
rhCol17 regulated the expressions of BM-related key molecules in photoaging HaCaTs. **(A)** Relative mRNA expression levels of COL4A1, COL7A1, COL17A1, LAMB3, and MMP2 determined by qRT-PCR. **(B)** Representative blots of COL4A1, COL7A1, COL17A1, LAMB3, MMP2, and corresponding internal reference GAPDH determined by Western blot. **(C)** Quantitative analysis of optical density in Western blot. N = 3. *P < 0.05, **P < 0.01, ***P < 0.001, ****P < 0.0001, ns, no significant.

Compared with the control, BL exposure significantly increased intracellular ROS levels and the proportion of SA-β-gal positive cells, while rhCol17 pretreatment significantly reduced both ROS accumulation and cellular senescence (Figures 1C,D). We next assessed the effects on inflammatory factors. The transcriptional and secreted levels of IL-1β, IL-6, and TNF-α were significantly increased after BL exposure compared with the control, whereas rhCol17 pretreatment effectively attenuated these increases (Figures 1E,F). Scratch-wound assays showed that the scratch closure area of HaCaTs was significantly reduced in the BL group at 24 and 48 h, whereas the BL + rhCol17 group exhibited significantly enhanced scratch closure compared with the BL group (Figures 1G,H). Transwell assays demonstrated that rhCol17 pretreatment significantly increased the number of migrated HaCaTs following BL irradiation (Figures 1I,J). We further examined the expression of key molecules associated with the functions of BM. BM integrity is essential for maintaining epidermal adhesion and barrier homeostasis. The mRNA and protein levels of COL4A1, COL7A1, COL17A1, and LAMB3 were significantly decreased after BL exposure. MMP2, which degrades type IV collagen and is abundantly expressed in human skin, is closely associated with DNA damage–induced photoaging in HaCaTs (Kim et al., 2022). Its levels were significantly upregulated by BL irradiation. Importantly, rhCol17 pretreatment suppressed BL-induced increases in the mRNA and protein expressions of these molecules (Figures 2A–C).

Collectively, these results demonstrated that rhCol17 mitigates the adverse effects of BL on epidermal proliferation, oxidative stress, senescence, inflammation, migration, and the expression of structural molecules, which are key features of photoaging.

### 3.2 rhCol17 attenuated BL-induced photoaging in dermal fibroblasts

**FIGURE 3:**
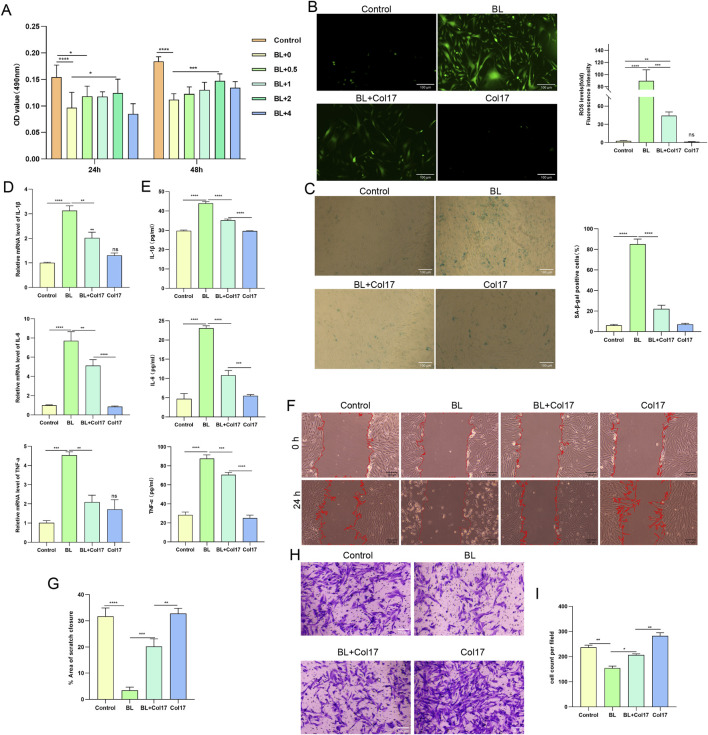
rhCol17 alleviated BL-induced photoaging in HDFs. **(A)** Cell viability assessed by CCK8 assay at 24 and 48 h after BL exposure with or without rhCol17 pretreatment at the indicated concentrations. **(B)** Representative fluorescence images of cellular ROS levels measured using DCFH-DA assay (scale bar = 100 μm, left). From left to right: control, BL exposure group, BL + rhCol17 group (BL exposure with 2 mg/mL rhCol17 pretreatment), and Col17 group (rhCol17 only). Quantitative analysis of ROS fluorescence intensity (light). **(C)** Representative images of SA-β-gal staining (scale bar = 100 μm, left). Quantitative analysis of SA-β-gal staining (right). **(D)** Relative mRNA expression levels of IL-1β, IL-6, and TNF-α determined by qRT-PCR. **(E)** Concentrations of IL-1β, IL-6, and TNF-α in the culture supernatants measured by ELISA. **(F)** Representative images of scratch assays at 24 h (scale bar = 300 μm). **(G)** Quantitative analysis of scratch closure area (%) in the scratch assay. **(H)** Representative images of transwell migration assays. **(I)** Quantitative analysis of the migrated cell count per field. N = 3. *P < 0.05, **P < 0.01, ***P < 0.001, ****P < 0.0001. NS, no significant vs. control group.

BL can penetrate up to approximately 1 mm into the skin, reaching the superficial dermis where fibroblasts are abundant (Sadowska et al., 2021). To determine whether rhCol17 exerts protective effects on dermal fibroblasts, HDFs were exposed to BL with or without rhCol17 pretreatment. Similar to the findings in HaCaT cells, a concentration of 2 mg/mL rhCol17 showed the most effective protection against BL-induced cytotoxicity (Figure 3A). Compared with the control group, BL exposure significantly increased intracellular ROS levels (Figure 3B), the proportion of SA-β-gal–positive cells (Figure 3C), and the transcription and secretion levels of inflammatory cytokines (Figures 3D,E), while reducing scratch closure and migration capacity (Figures 3F–I). Pretreatment with rhCol17 significantly alleviated these effects.

**FIGURE 4:**
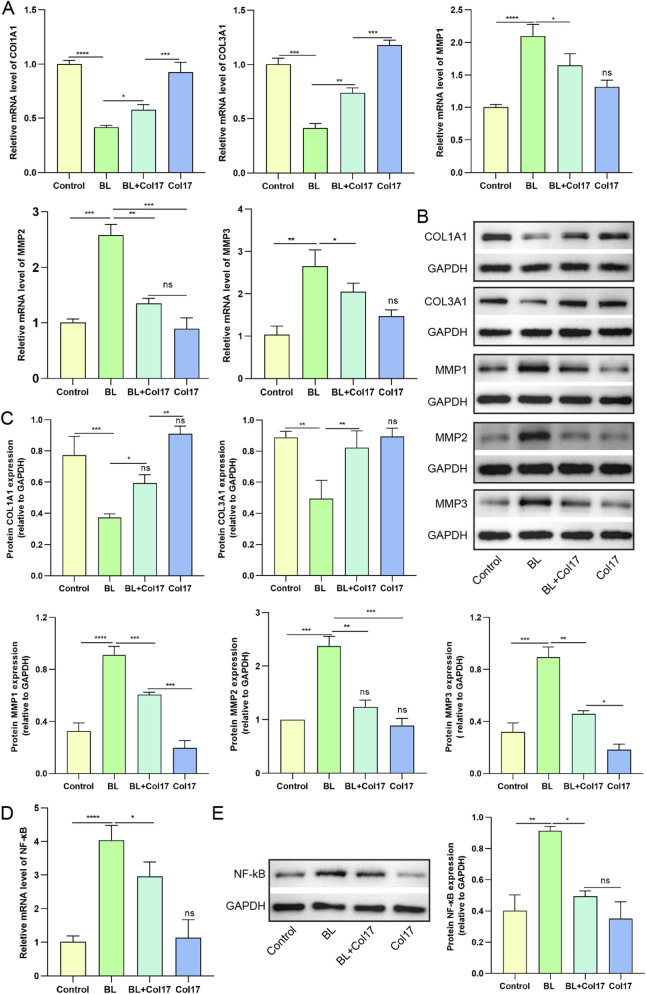
rhCol17 regulated the expressions of extracellular matrix-related key molecules in photoaging HDFs. **(A)** Relative mRNA expression levels of COL1A1, COL3A1, MMP1, MMP2, and MMP3 determined by qRT-PCR. **(B)** Representative blots of COL1A1, COL3A1, MMP1, MMP2, MMP3, and corresponding internal reference GAPDH determined by Western blot. **(C)** Quantitative analysis of optical density in Western blot. **(D)** Relative mRNA expression levels of NF-κB. **(E)** Representative blots (left) and quantification (right) of NF-κB. N = 3. *P < 0.05, **P < 0.01, ***P < 0.001, ****P < 0.0001, ns, no significant.

We next examined the effects of rhCol17 on collagen molecules and MMPs, which are essential for ECM homeostasis, and whose dysregulation is closely associated with photoaging (Liu et al., 2025). BL irradiation markedly decreased the mRNA and protein levels of COL1A1 and COL3A1, while significantly increasing those of MMP1, MMP2, and MMP3 (Figures 4A–C). Treatment with rhCol17 mitigated these BL-induced alterations at both mRNA and protein levels. Persistent activation of NF-κB has been reported to promote inflammation and upregulate MMP expression, thereby contributing to photoaging (Wei et al., 2025). Consistent with these findings, BL exposure significantly activated NF-κB signaling in HDFs, whereas rhCol17 pretreatment effectively inhibited this activation (Figures 4D,E).

Taken together, these results indicated that rhCol17 mitigates BL-induced dermal photoaging by enhancing fibroblast proliferation and migration, reducing ROS accumulation, cellular senescence, and inflammation, and modulating the expression of collagen, MMPs, and NF-κB.

### 3.3 rhCol17 attenuated BL-induced skin photoaging in a rat model

**FIGURE 5:**
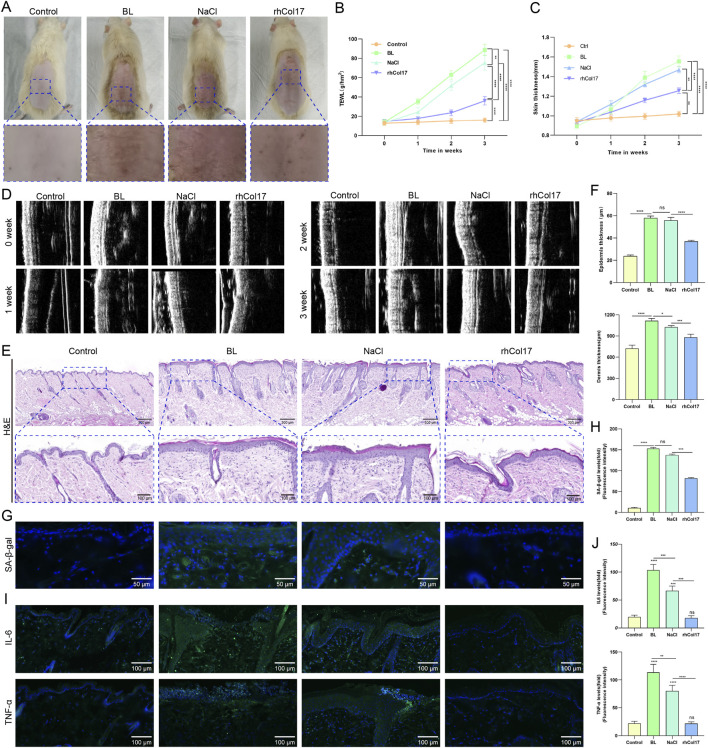
rhCol17 attenuated BL-induced skin photoaging in a rat model. **(A)** Representative gross images and corresponding enlarged views of dorsal skin. From left to right: control, BL, NaCl (BL exposure with microneedle-assisted saline application), and rhCol17 (BL exposure with microneedle-assisted rhCol17 application) groups. **(B)** Quantitative analysis of transepidermal water loss (TEWL) at weeks 0, 1, 2, and 3. **(C)** Quantitative analysis of skin thickness measured by ultrasound at weeks 0, 1, 2, and 3. **(D)** Representative ultrasound images of dorsal skin thickness at weeks 0, 1, 2, and 3. **(E)** Representative H&E staining images (upper: scale bar = 300 μm; lower: scale bar = 100 μm). **(F)** Quantification of epidermal and dermal thickness. **(G)** Representative fluorescence images of SA-β-gal staining. **(H)** Quantitative analysis of SA-β-gal fluorescence intensity. **(I)** Representative fluorescence images of IL-6 and TNF-α staining. **(J)** Quantitative analysis of IL-6 and TNF-α fluorescence intensity. N = 4. *P < 0.05, **P < 0.01, ***P < 0.001, ****P < 0.0001, ns, no significant.

To evaluate the protective effects of rhCol17 against BL induced photoaging *in vivo*, a rat photoaging model was established. After continuous BL exposure for 3 weeks, the dorsal skin of rats exhibited marked dryness and increased wrinkling, confirming successful model induction. In contrast, rats treated topically with rhCol17, but not NaCl, displayed visibly smoother skin (Figure 5A). TEWL and skin thickness were measured weekly to assess barrier integrity and skin condition. Both the BL and NaCl groups showed severe water loss and significant skin thickening, whereas rhCol17 treatment significantly reduced TEWL and attenuated skin thickening (Figures 5B–D). Histological examination with H&E staining revealed pronounced thickening of both the epidermis and dermis in the BL and NaCl groups (Figures 5E,F). The dermal connective tissue appeared loose, with disorganized collagen bundles and enlarged inter-fibrillar spaces, indicating structural disruption. In contrast, rats in rhCol17 group exhibited reduced epidermal and dermal thickness and a skin architecture closer to that of the control group. Immunofluorescence analysis showed that the fluorescence intensities of SA-β-gal (Figures 5G,H), IL-6, and TNF-α (Figures 5I,J) were significantly increased in the BL group, suggesting enhanced cellular senescence and inflammation. Compared with the NaCl group, rhCol17 group significantly reduced their fluorescence intensities. These results indicated that rhCol17 effectively counteracts BL-induced skin aging, inflammation, and structural damage in rat models.

**FIGURE 6:**
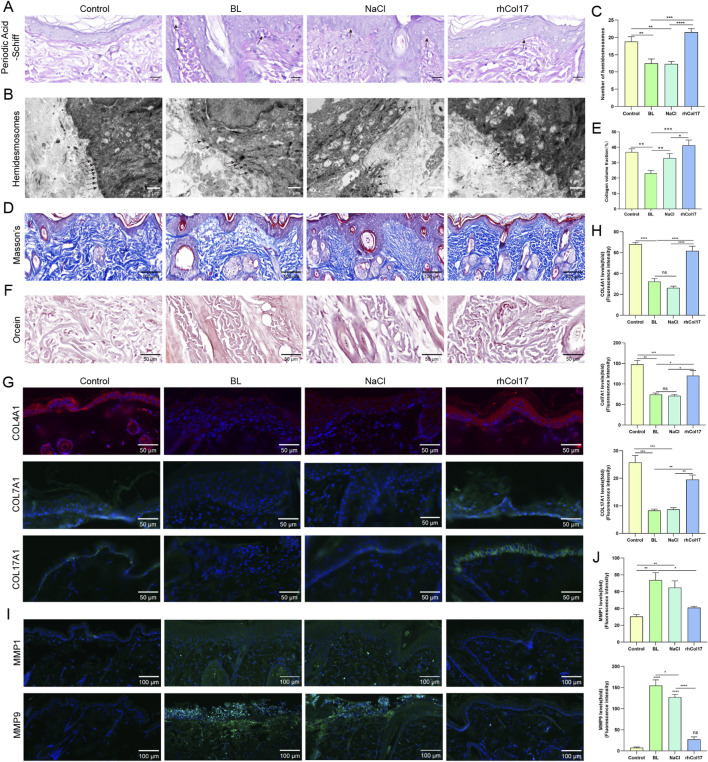
Histological and immunofluorescence analyses of the dorsal skin in photoaging rats. **(A)** Representative PAS staining images. **(B)** Representative transmission electron microscopy images. Arrows indicate hemidesmosomes. **(C)** Quantitative analysis of hemidesmosome numbers based on TEM results. **(D)** Representative Masson’s trichrome staining images. **(E)** Quantitative analysis of collagen volume fraction from Masson’s trichrome staining. **(F)** Representative Orcein staining images of elastic fibers. **(G)** Representative immunofluorescence images of COL4A1, COL7A1, and COL17A1. **(H)** Quantitative analysis of COL4A1, COL7A1, and COL17A1 fluorescence intensity. **(I)** Representative immunofluorescence images of MMP1 and MMP9. **(J)** Quantitative analysis of MMP1 and MMP9 fluorescence intensity. N = 4. *P < 0.05, **P < 0.01, ***P < 0.001, ****P < 0.0001, ns, no significant.

To further assess BM integrity, PAS staining was performed (Figure 6A). Rats in BL and NaCl groups exhibited vacuolar degeneration and focal discontinuities within the BM zone, whereas the BM structure remained largely intact in the rhCol17 group. Transmission electron microscopy confirmed these findings, revealing BM discontinuity and a reduced number of hemidesmosomes after BL exposure, while rhCol17 treatment preserved hemidesmosome number and distribution (Figures 6B,C). Dermal collagen and elastic fibers, key components for skin elasticity and structural support, were visualized using Masson’s trichrome and Orcein staining (Figures 6D–F). Compared with the control group, BL and NaCl treatment caused a marked reduction in collagen deposition, elastic fiber degeneration, and focal aggregation, consistent with fragmentation and loss of integrity. In contrast, the rhCol17-treated group exhibited denser collagen fibers and well-organized elastic structures without obvious breakage. Finally, we examined the expression of molecules associated with BM integrity and ECM homeostasis. Immunofluorescence staining revealed that the levels of COL4A1, COL7A1, and COL17A1 were markedly decreased (Figures 6G,H), while MMP1 and MMP9 were elevated after BL exposure (Figures 6I,J). rhCol17 treatment restored these protein levels toward normal values.

Collectively, these results demonstrated that BL irradiation disrupts skin architecture, damages the BM zone and dermal matrix fibers, and causes dysregulation of key structural molecules. rhCol17 treatment effectively alleviates these alterations, preserving BM integrity, ECM stability, and overall skin homeostasis.

### 3.4 rhCol17 alleviated BL-induced photoaging in keratinocytes by downregulating Notch1 signaling

**FIGURE 7:**
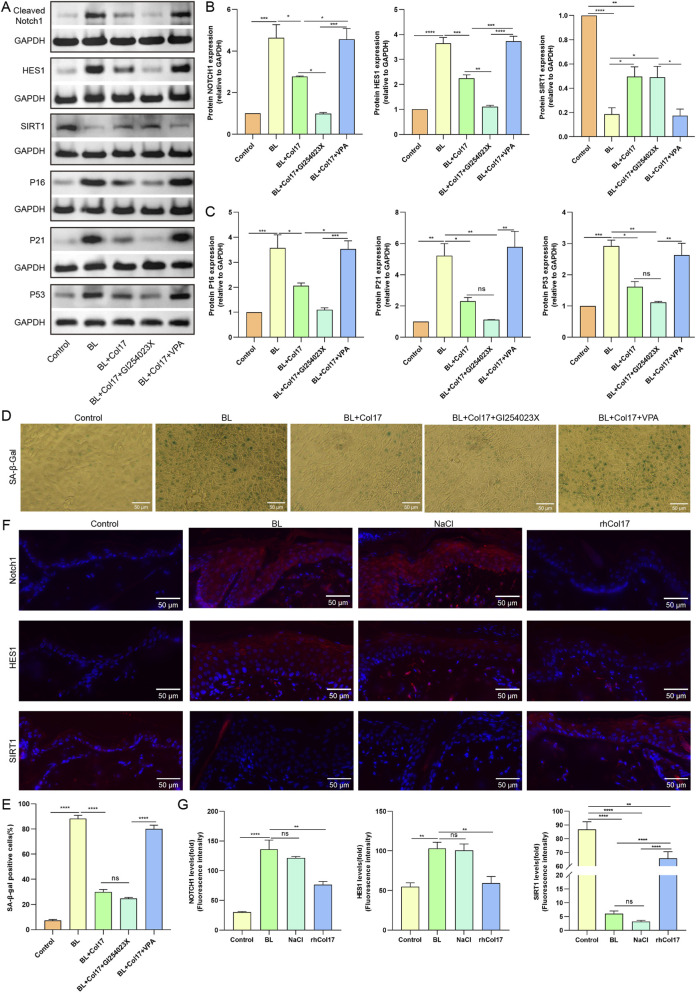
rhCol17 alleviated BL-induced photoaging in HaCaTs by downregulating Notch1 signaling. **(A)** Representative blots of Notch1, HES1, SIRT1, P16, P21, P53, and corresponding internal reference GAPDH determined by Western blot. **(B, C)** Quantitative analysis of optical density in Western blot. **(D)** Representative images of SA-β-gal staining. **(E)** Quantitative analysis of SA-β-gal staining. **(F)** Representative fluorescence images of IL-6 and TNF-α staining. **(G)** Quantitative analysis of Notch1, HES1, and SIRT1 fluorescence intensity. N = 3. *P < 0.05, **P < 0.01, ***P < 0.001, ****P < 0.0001, ns, no significant.

Notch1 plays a critical role in regulating keratinocyte differentiation and self-renewal and has been implicated in photoaging processes (Nguyen et al., 2006; Zhang H. et al., 2024). To investigate whether rhCol17 exerts its protective effects through the Notch pathway, we examined the expression of Notch-related signaling molecules in BL-exposed HaCaTs. Western blot analysis revealed that BL irradiation significantly increased the protein levels of cleaved Notch1, HES1, p16, p21, and p53, while SIRT1 expression was significantly decreased (Figures 7A–C). Pretreatment with rhCol17 partially restored these changes. Furthermore, inhibition of Notch activation using GI254023X, an ADAM10 inhibitor required for Notch cleavage, completely reversed these alterations to levels comparable to the control. Conversely, co-treatment with the Notch activator VPA abolished the regulatory effects of rhCol17 on these signaling molecules.

To further verify the involvement of Notch signaling in rhCol17-mediated protection, we examined SA-β-gal activity in HaCaTs after BL and rhCol17 treatment (Figures 7D,E). GI254023X enhanced the anti-photoaging effects of rhCol17, whereas VPA treatment led to significant accumulation of SA-β-gal–positive cells, indicating that Notch activation counteracted the protective effects of rhCol17 against BL-induced senescence.

Consistent with the *in vitro* findings, immunofluorescence staining of rat skin revealed that BL exposure significantly increased the fluorescence intensity of Notch1 and HES1, but reduced SIRT1 levels in the epidermis (Figures 7F,G). rhCol17 treatment restored these protein expression patterns toward those of the control group.

Taken together, these results suggested that rhCol17 protects keratinocytes from BL-induced photoaging by downregulating Notch1 signaling.

## 4 Discussion

Different wavelengths of visible light exert distinct beneficial or detrimental effects on the skin (Pourang et al., 2022). With increasing exposure to BL from solar radiation and digital electronic devices, BL has received growing attention in recent years. Prolonged or high-dose exposure to BL has been shown to induce cellular dysfunction and skin barrier impairment, ultimately leading to skin aging and collagen loss (Das et al., 2023; Habib et al., 2024). In parallel, recombinant collagens derived from human genes have emerged as promising biomaterials due to their favorable safety profiles and biological functionality, offering potential benefits in skin repair and regeneration (Wang et al., 2022). In this study, we employed a BL-induced photoaging model to evaluate the protective effects of rhCol17.

Keratinocytes constitute over 80% of epidermal cells and play essential roles in skin protection, immune regulation, and tissue repair (Piipponen et al., 2020), whereas fibroblasts are the predominant cell type in the dermis and are responsible for synthesizing and remodeling ECM components, including collagen and elastic fibers, thereby maintaining skin elasticity and structural integrity (Zhang J. et al., 2024). BL has been shown to modulate cellular biological functions in a dose-dependent manner. For instance, BL at doses ranging from 20.6 to 41.2 J/cm^2^ significantly inhibits keratinocyte and fibroblast proliferation and migration (Rossi et al., 2021). Consistently, our results showed that 20 J/cm^2^ BL exerted inhibitory effects *in vitro*, which were significantly alleviated by rhCol17 treatment. Beyond phenotypic alterations, BL exposure also induces transcriptional and protein-level changes in skin cells (Shao et al., 2025; Tonolli et al., 2025). MMPs are key mediators of photoaging, with MMP1, MMP2, MMP3, and MMP9 reported to be upregulated in fibroblasts following UV irradiation. Elevated MMP expression and reduced LAMB3 levels contribute to BM disruption and elastic fiber degradation, thereby accelerating skin aging (Amano, 2016). Moreover, photoaging is associated with decreased expression of multiple collagen genes, including COL1A1, COL3A1, COL4A1, and COL7A1 (Cheong et al., 2018). In our study, BL exposure induced dysregulation of these molecules at both the transcriptional and protein levels, whereas rhCol17 treatment partially restored their expression. Collectively, preliminary *in vitro* experiments indicated that rhCol17 may prevent damage to epidermal and dermal cells by inhibiting ECM-degrading enzymes and enhancing the synthesis of essential collagen and BM components, thereby reducing photoaging induced by BL.

Based on previous studies, we established a BL-induced photoaging animal model using a cumulative dose of 120 J/cm^2^ (Lim et al., 2021; Ge et al., 2023). Photoaged skin typically presents with dryness, scaling, epidermal thickening, and increased wrinkle formation (Shin et al., 2019). Consistent with these features, skin physiological assessment, ultrasound imaging, and SA-β-gal staining confirmed successful model establishment. Unlike intrinsic aging, which is characterized by epidermal thinning, photoaging is frequently associated with epidermal thickening due to impaired degradation of desmosomes between keratinocytes (Sabu et al., 2024). In addition, our data showed an increase in dermal thickness following BL exposure. This finding was accompanied by elastin degeneration and collagen fiber degradation with a loosened and disorganized architecture, which may contribute to abnormal dermal thickening during photoaging (Hua et al., 2014). Similar to the photoaging induced by UV radiation, the *in vivo* model in our study showed that BL exposure causes a significant loss of collagen in the back skin of rats, along with a notable increase in MMPs. This disruption may disturb the balance of the ECM, ultimately affecting the skin’s structure and function (Zhao et al., 2019; Dong et al., 2026). The protective effect of rhCol17 is further demonstrated by its ability to enhance the structure and function of the rats’ back skin, as well as regulate collagen and MMP expression at the protein level. COL17 is a critical component of hemidesmosomes, which are essential for epidermal-dermal adhesion, regulation of cell polarity, proliferation, and migration (Hiroyasu and Tsuruta, 2022). Reduced hemidesmosome density has been reported in aged skin and in individuals exposed to UV radiation, resulting in compromised anchorage of basal keratinocytes (Coelho et al., 2015; Roig-Rosello et al., 2024). Our TEM analysis demonstrated a significant reduction in hemidesmosome numbers in BL-induced photoaged rat skin, which further supports the pivotal role of hemidesmosomes in skin aging. Furthermore, rhCol17 treatment restored hemidesmosome density to near-control levels. Overall, we speculated that rhCol17 not only protects epidermal and dermal cells from BL-induced molecular damage and disruption of functional homeostasis, but may also serve as an anchoring scaffold that contributes to the maintenance of epidermal-dermal structural integrity by modulating hemidesmosomes.

Excessive ROS generation and inflammatory activation are hallmark features of photoaging. ROS accumulation induces oxidative damage to cellular components, promotes degradation of structural proteins, and ultimately compromises dermal integrity and function (Zhang et al., 2025). Moreover, ROS-triggered inflammatory signaling amplifies skin damage and can propagate senescence to neighboring cells through bystander effects involving ROS and pro-inflammatory cytokines (Salminen et al., 2022). In this study, rhCol17 significantly attenuated ROS accumulation and suppressed the expression of inflammatory cytokines, including IL-6, IL-1β, and TNF-α, suggesting its potential antioxidative and anti-inflammatory properties in preventing BL-induced photoaging.

Dysregulation of intracellular signaling pathways represents another critical mechanism underlying BL–induced skin damage. BL-generated ROS have been shown to activate Nrf2 and MAPK signaling pathways, thereby promoting oxidative stress responses (Liu et al., 2023). Our data further revealed activation of the Notch signaling pathway in keratinocytes following BL exposure. HES1, a key downstream effector of Notch1, promotes cellular senescence through upregulation of the p53/p21/p16 axis, whereas SIRT1 activation counteracts this process (Wang et al., 2019; Duan et al., 2022). The rhCol17-induced modulation of these molecules supports the involvement of Notch signaling in its protective effects against BL-induced photoaging. Additionally, activation of NF-κB was observed in HDFs. As previously reported, ROS can enhance the expression of pro-inflammatory factors through NF-κB activation, thereby promoting the senescence-associated secretory phenotype (Wang et al., 2019). In the present study, rhCol17 significantly reduced ROS production in HDFs and suppressed NF-κB activation as well as downstream inflammatory cytokines. These findings suggest that rhCol17 may regulate NF-κB signaling through an anti-inflammatory mechanism and consequently inhibit the release of pro-inflammatory mediators. COL17 and integrin α6β4 are key transmembrane proteins involved in cell–matrix adhesion in the skin. Previous research has shown that disruption of COL17 or ITGB4 leads to similar dysregulation of ECM and focal adhesion–related gene networks, which are associated with wound healing processes and inflammatory responses (Wang Y. et al., 2025). Moreover, integrin α6β4 has been reported to activate both Notch and NF-κB signaling pathways in certain cellular contexts in cancer cells (Friedland et al., 2007; Hao et al., 2022). Therefore, these findings raise the possibility that integrin α6β4 may serve as a potential signaling hub mediating the effects of rhCol17 in both keratinocytes and fibroblasts. Further studies investigating the crosstalk between epidermal and dermal signaling pathways will be important for elucidating the mechanisms by which rhCol17 contributes to skin homeostasis and protects against photoaging-related damage.

Considering the absorption efficiency, metabolic processes, and potential dynamic clearance mechanisms *in vivo* (Lai et al., 2026), a higher concentration of rhCol17 was used in the animal experiments to ensure sufficient local bioavailability in skin tissues. Although our study provides new evidence supporting the anti-photoaging effects of rhCol17, several limitations should be acknowledged. First, rhCol17 was delivered using microneedle-assisted transdermal administration in the animal model. This approach was chosen based on both the molecular characteristics of rhCol17 and the barrier function of the stratum corneum (Trommer and Neubert, 2006). The therapeutic target of COL17 in barrier repair is primarily located at the epidermal basement membrane and the superficial dermis. Microneedle-assisted delivery has been reported to enhance drug diffusion and improve local tissue penetration, and inhibit inflammatory responses (Jie et al., 2025). However, the therapeutic effects observed in this group should also be interpreted with consideration of the potential non-specific repair effects associated with microneedle-assisted NaCl treatment. Previous studies have shown that NaCl (physiological saline) is generally considered inert and does not exert therapeutic effects on photoaging (Zhou et al., 2020; Wang et al., 2022). In our study, the microneedle-assisted NaCl group showed modest improvements mainly in TEWL, inflammatory markers (IL-6 and TNF-α), and collagen volume fraction. These changes are consistent with previously reported effects of microneedle-induced mechanical stimulation, which can promote the release of reparative growth factors and stimulate collagen synthesis (Hamed et al., 2024). Second, the present study lacked comparisons with positive control agents. Future studies should compare the anti-BL photoaging effects of rhCol17 with clinically used anti-photoaging treatments to further evaluate its therapeutic advantages and enhance its potential for clinical translation.

## 5 Conclusion

Our study investigated the protective effects of rhCol17 against BL-induced photoaging using both *in vitro* and *in vivo* models. Our results suggested that rhCol17 could protect epidermal and dermal cells from BL-induced molecular damage and maintain functional homeostasis by attenuating ROS accumulation, suppressing inflammatory responses, and reducing MMPs production, while simultaneously promoting the expression of key collagen and BM components. Furthermore, rhCol17 enhanced hemidesmosome density, which may contribute to the maintenance of epidermal-dermal adhesion. Mechanistically, we identified the Notch signaling pathway as a critical mediator in keratinocytes underlying the anti-photoaging effects of rhCol17. Collectively, these findings highlight the potential of rhCol17 as a promising therapeutic agent for preventing BL-related skin damage and preserving skin structural and functional integrity.

## Data Availability

The original contributions presented in the study are included in the article/Supplementary Material, further inquiries can be directed to the corresponding authors.
