# Supplementary material for: Therapeutic potential of recombinant human collagen XVII in blue light-induced skin photoaging: preserving epidermal-dermal structural integrity and functional homeostasis
Source: Front Bioeng Biotechnol. 2026 Apr 28;14:1806274. doi: 10.3389/fbioe.2026.1806274 (PMC13161066; doi:10.3389/fbioe.2026.1806274)
Supplement: Supplementary file 1 [file Table1.docx]

**Table S1. Primer sequence of target genes.**

| **Gene name** | **Primer sequence** |
| --- | --- |
| IL-1β-F | CCAAACCTCTTCGAGGCACA |
| IL-1β-R | GCTGCTTCAGACACTTGAGC |
| IL-6-F | CCTTCTCCACAAGCGCCTTC |
| IL-6-R | GGAAGGCAGCAGGCAACA |
| TNF-α-F | GCCCATGTTGTAGCAAACCC |
| TNF-α-R | TGAGGTACAGGCCCTCTGAT |
| COL4A1-F | TAGACGGATATCGGGGGCCT |
| COL4A1-R | TGGATTTGAAAAAGCAATGGCACT |
| COL7A1-F | GATGGAGTGCCTGGTATCCG |
| COL7A1-R | GGGACCAGCTTCTCCCTTG |
| COL17A1-F | CAGGACCTGTCACCACCATC |
| COL17A1-R | CGTACCCCGAAGTCCGTAAG |
| LAMB3-F | CTCTTGTGTTTTGCCCTGCC |
| LAMB3-R | GTCTCAGGCTTGGTCAGTCC |
| MMP2-F | TACAGGATCATTGGCTACACACC |
| MMP2-R | GGTCACATCGCTCCAG |
| MMP1-F | TTGTCCCGATGATCTCCCCT |
| MMP1-R | TTGTCCCGATGATCTCCCCT |
| MMP3-F | CACAGACCTGACTCGGTTCC |
| MMP3-R | GAGTCAGGGGGAGGTCCATA |
| COL1A1-F | CCCCGAGGCTCTGAAGGT |
| COL1A1-R | GCAATACCAGGAGCACCATTG |
| COL3A1-F | TAAAGGCGAAATGGGTCCCG |
| COL3A1-R | GGCACCATTCTTACCAGGCT |
| NF-κB-F | ACTCGCCACCCGGCTTC |
| NF-κB-R | GTGCCATCTGTGGTTGAAATACT |
| GAPDH-F | GGAGCGAGATCCCTCCAAAAT |
| GAPDH-R | GGCTGTTGTCATACTTCTCATGG |
