# Supplementary figures and images for: Therapeutic potential of recombinant human collagen XVII in blue light-induced skin photoaging: preserving epidermal-dermal structural integrity and functional homeostasis
Source: Front Bioeng Biotechnol. 2026 Apr 28;14:1806274. doi: 10.3389/fbioe.2026.1806274 (PMC13161066; doi:10.3389/fbioe.2026.1806274)

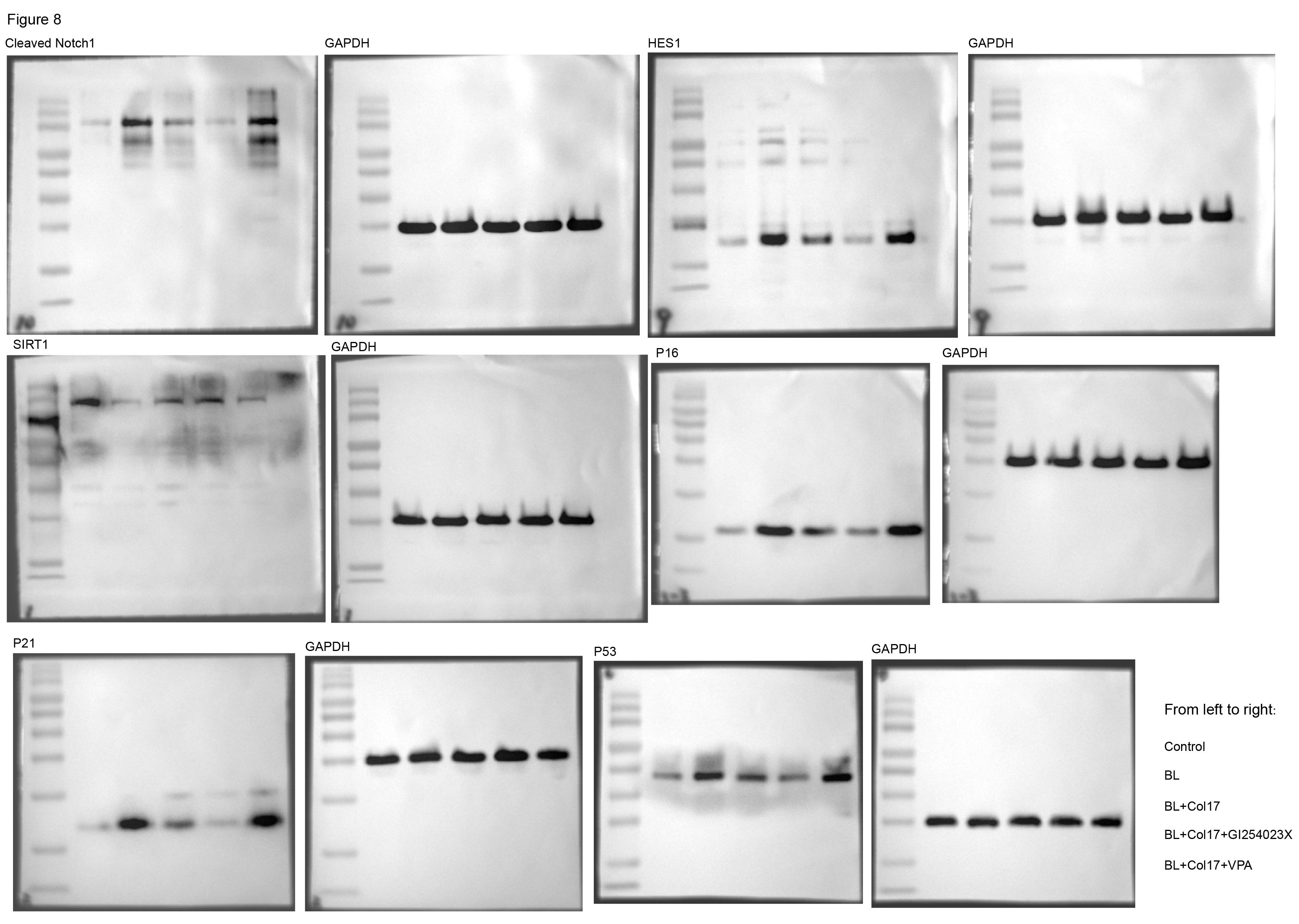

Supplement: Supplementary file 2 [file Image3.tif]

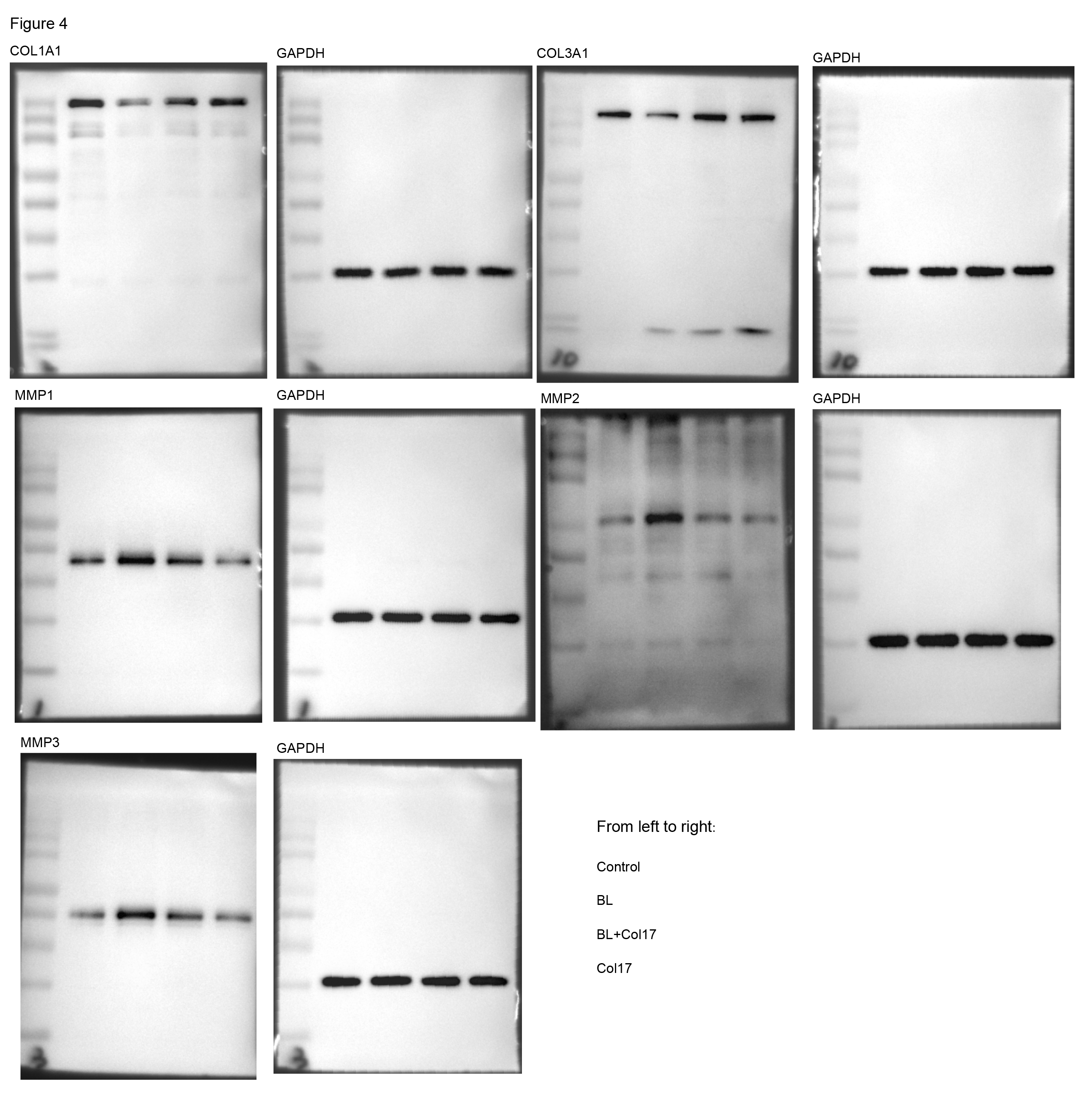

Supplement: Supplementary file 3 [file Image2.tif]

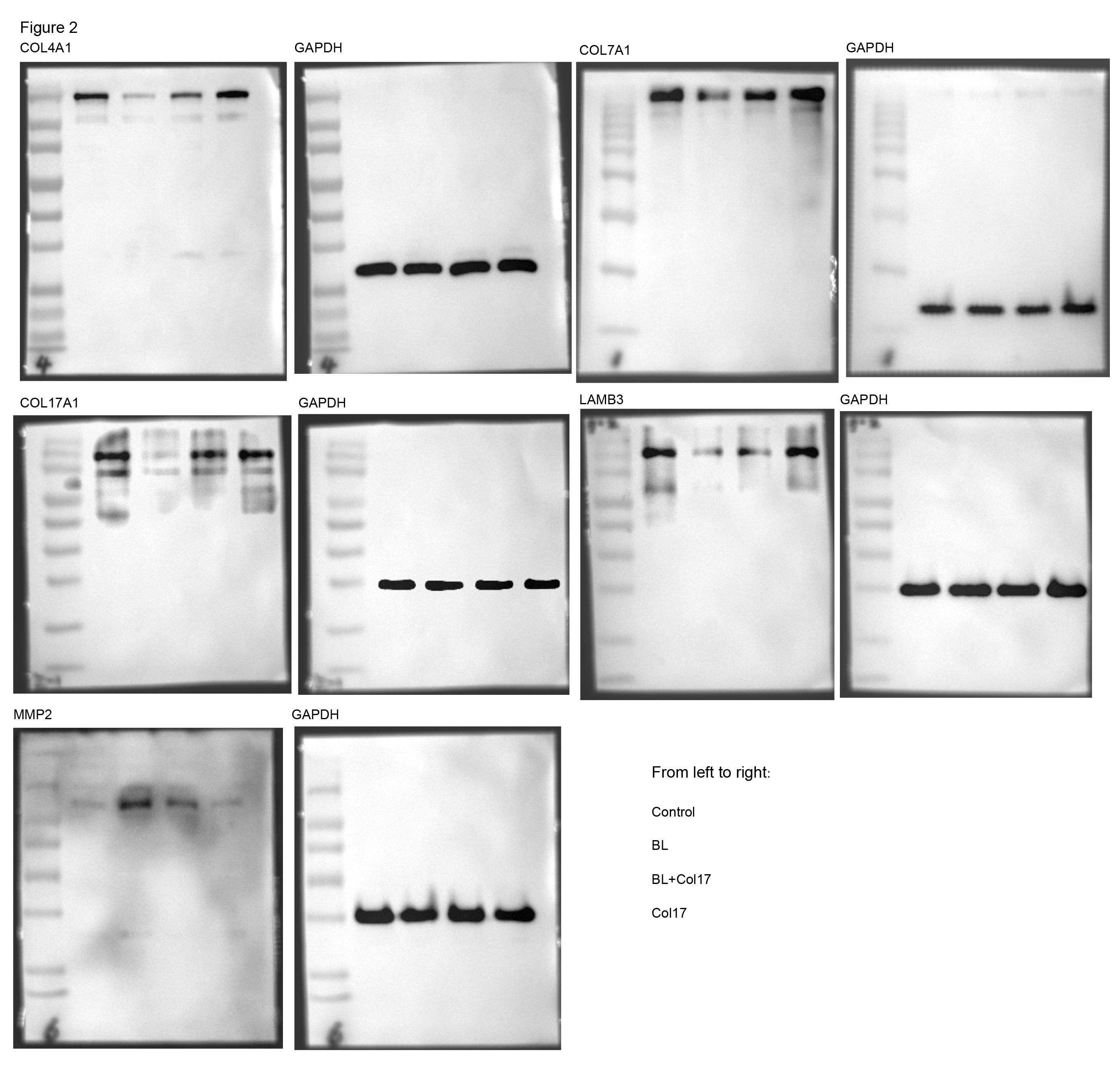

Supplement: Supplementary file 4 [file Image1.tif]
